# Genetic susceptibility shaped by biological state: beyond gene-environment interaction

**DOI:** 10.3389/fgene.2026.1877629

**Published:** 2026-06-25

**Authors:** Hui-Qi Qu, Hakon Hakonarson

**Affiliations:** 1 The Center for Applied Genomics, Children’s Hospital of Philadelphia, Philadelphia, PA, United States; 2 Department of Pediatrics, The Perelman School of Medicine, University of Pennsylvania, Philadelphia, PA, United States; 3 Division of Human Genetics, Children’s Hospital of Philadelphia, Philadelphia, PA, United States; 4 Division of Pulmonary Medicine, Children’s Hospital of Philadelphia, Philadelphia, PA, United States; 5 Faculty of Medicine, University of Iceland, Reykjavik, Iceland

**Keywords:** biological state, clinical translation, disease mechanism, gene-environment interaction, genetic susceptibility, multi-omics

## Abstract

This mini-review proposes a state-aware approach for interpreting gene-environment interaction (G × E). Biological state is the internal condition in which genetic susceptibility becomes functional. A measured exposure may precede that state, reflect it, incompletely measure internal dose or response, or trigger transition into it. A genotype-by-exposure interaction may therefore be statistically valid but mechanistically ambiguous when the measured exposure does not identify the condition that shapes susceptibility. Genetic effects on gene regulation may appear only after immune stimulation, within specific cell states, or in diseased tissue, supporting biological state as a condition of variant function. At the individual level, susceptibility may emerge during infection, puberty, insulin resistance, inflammatory activation, hormonal transition, or biological ageing. State-aware analysis requires investigators to define what the exposure represents in relation to host biology, measure the relevant state with spatial and temporal specificity, and evaluate replication across comparable biological contexts. This approach moves G × E interpretation from exposure-defined interaction toward biological-state precision in genetic epidemiology.

## Introduction

Genetic susceptibility is usually treated as a genetic property that remains constant across the life course. In the conventional gene-environment interaction (G × E) model, genetic effects differ across exposure contexts, and exposure effects differ across genotypes. This framework was important because it made genetic risk conditional rather than deterministic (Hunter, 2005). However, it leaves a central problem unresolved: the same genotype and the same type of exposure do not necessarily produce the same clinical or biological outcome.

**FIGURE 1:**
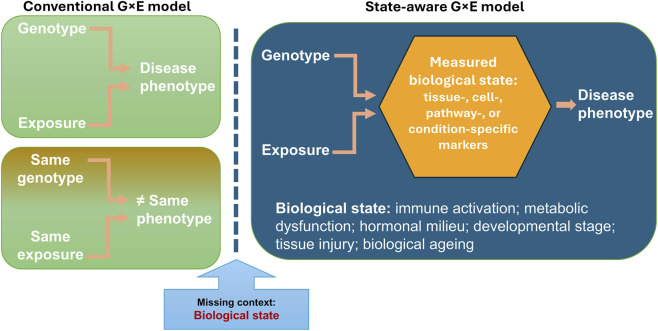
Biological state as the missing context in gene-environment interaction. The conventional G × E model treats genotype and exposure as joint determinants of disease phenotype. This model does not explain why the same genotype and the same exposure category may fail to correspond to the same clinical or biological outcome. The central barrier is the missing biological state context. In the state-aware model, biological state is the internal condition in which genetic susceptibility becomes expressed. Biological state can be measured by tissue-, cell-, pathway-, or condition-specific markers and may shape whether, when, and how genetic susceptibility is translated into disease.

As a perspective-oriented synthesis rather than a comprehensive mechanistic review, this mini-review proposes that biological state may represent a critical missing context in interpreting G × E (Figure 1). Biological state is the internal condition in which genetic susceptibility becomes functional. It is broader than a mediator or effect modifier because its analytic role depends on timing and measurement. A state may carry the effect of an exposure, change the effect of genotype, capture internal dose or response, or signal preclinical disease. It is also distinct from a phenotype or endophenotype because it is used to define the condition in which susceptibility is expressed, not only the trait being explained. Biological state can be measured by markers of the relevant tissue, cell type, pathway, or clinical condition. An exposure variable may serve as a proxy for the internal condition in which susceptibility becomes functional. The critical question is whether the exposure variable identifies the biological state in which genetic susceptibility is translated into disease.

Biological state extends existing approaches by focusing on the internal condition that determines DNA variant function and disease penetrance. For example, exposomics emphasizes that the genome must be complemented by systematic exposure measurement (Wild, 2005), but exposure factors alone may not define the underlying biological state when the focus remains on the external environment. Context-specific eQTL studies can reveal regulatory variant effects in specific cell types or activation states that are not detectable at baseline (Fairfax et al., 2014), but an eQTL observed in one tissue or state may not be relevant to the disease being studied. Causal mediator-versus-modifier frameworks clarify whether an intermediate variable carries an exposure effect or changes a genetic effect (Feinberg et al., 2016), but biological state may act as an on-off switch that determines whether mediation or modification is active (e.g., HPV infection in cervical cancer). The state-aware framework brings these scientific levels together into a systematic analytic algorithm. It is an approach to investigate the internal condition in which genetic susceptibility becomes penetrant, without being limited to a single scientific branch.

## Measured exposures and hidden biological state

Dietary intake illustrates why measured exposures need biological context. Low red meat intake may reflect intentional health behavior, reduced appetite, early disease activity, organ dysfunction, or clinical dietary management. High vegetable intake may mark a health-conscious dietary pattern, socioeconomic context, or pre-existing illness affecting food tolerance. A genotype-by-diet interaction may therefore be statistically valid but biologically ambiguous when the exposure category does not identify the internal condition in which susceptibility is expressed. Precision environmental health frameworks emphasize that exposure measures require biological context before they can be interpreted mechanistically (Motsinger-Reif et al., 2024). Biological state adds to existing frameworks such as developmental programming, epigenetics, exposomics, and context-dependent gene regulation by emphasizing the internal condition in interpreting G × E analysis. At the conceptual level, it addresses why the same genotype and exposure category may not produce the same clinical or biological outcome: genetic susceptibility is translated into disease only within permissive biological states. An exposure may precede biological state, reflect it, incompletely measure the relevant internal response, or trigger transition into a new state.

### Exposure as upstream behavior

Some exposures reflect behaviors that precede disease and may plausibly modify risk. Dietary intake, physical activity, smoking, and medication use can all be meaningful upstream exposures. Yet even when the exposure is upstream, it may represent a broader behavioral pattern (e.g., diet clustering with physical activity, smoking with alcohol use, or medication use with healthcare access) rather than a single biological factor. Temporal order, spatial setting, and socioeconomic conditions can determine whether an upstream exposure acts as a determinant of biological state, a correlate of state, or a consequence of disease-related change. The exposure may also act indirectly or in parallel with other upstream processes.

Lifestyle can modify genetic risk, but lifestyle categories often combine behavior, physiology, and social context. Physical activity may reduce obesity risk through energy expenditure and improved insulin sensitivity, while also reflecting socioeconomic and built-environment conditions that determine whether regular activity is feasible, including neighborhood safety, walkability, work schedule, and leisure time (Kim et al., 2024; Polyzou and Polyzos, 2024). A genotype-by-physical-activity interaction may therefore reflect differences in energy expenditure, insulin sensitivity, or socioeconomic conditions correlated with activity. Stratification or adjustment for upstream socioeconomic context can help distinguish activity-related effects from correlated social conditions.

### Exposure as a downstream consequence of biological state

A measured exposure can also be downstream of biological state. In the red meat example, diet is not simply an external modifier of genetic risk, but may mark early disease, organ dysfunction, or illness-related dietary adaptation, as in kidney disease (Sullivan and Rebholz, 2023). If the exposure is analyzed as if it precedes disease, reverse causation can distort etiologic interpretation. Lagged exposure definitions, exclusion of early events, and sensitivity analyses for reverse causation, such as removing cases diagnosed soon after exposure assessment, are needed before assigning a causal interpretation to the interaction (Rezende et al., 2022).

### Exposure as an imperfect measure of internal dose or response

A measured exposure is often an imperfect measure of the biologically relevant dose or response. A dietary variable may record reported intake without capturing absorption, metabolism, or nutritional status (Raiten et al., 2021). In infection, the same issue applies to immune response: infection status may indicate pathogen exposure or serologic evidence of infection, but not pathogen load, interferon signaling, inflammatory intensity, immune-cell composition, or resolution of the response. The relevant modifier may be the immune state in which the genetic effect becomes visible.

### Exposure as a trigger of biological-state transition

Some exposures matter because they move the individual into a biological state in which genetic susceptibility becomes active. Infection provides a relatively direct example: viral infection may trigger interferon signaling or sustained inflammation, and host genetic variation has been linked to differences in COVID-19 disease severity, progression and downstream consequences. In noncommunicable disease, the relevant state transition is often gradual rather than acute. Overnutrition or inactivity may move individuals toward insulin resistance, adipose inflammation, or hepatic fat accumulation. Puberty, pregnancy, menopause, tissue injury, or ageing-related stress may similarly alter endocrine, immune, metabolic, or repair programs. In obesity studies, physical activity attenuates the association between *FTO* variants and body mass index (BMI), supporting G × E (Ahmad et al., 2013; Kilpeläinen et al., 2011). Biologically, the more direct modifier may be the transition away from insulin resistance and inflammatory activation, rather than physical activity as an exposure.

## Biological states in G × E

Biological state is the internal context in which genetic variation and exposures affect phenotype. Genetic susceptibility may remain latent in one state but become functional in another. At the molecular level, cellular state determines whether genetic variants are read through particular regulatory programs. At the organismal level, developmental stage, sex and hormonal milieu, metabolic-immune condition, and ageing-related tissue reserve determine the broader biological context in which susceptibility is expressed. Each state can alter when, where, or how genetic susceptibility is expressed through specific molecular mechanisms (Table 1).

**TABLE 1:** Molecular mechanisms underlying biological-state conditioning of genetic susceptibility.

Biological state	State-defining molecular logic	How genetic susceptibility is conditioned
Cellular activation state	Stimulation opens otherwise silent enhancers, recruits pathway-specific transcription factors, and engages non-coding RNA regulation	Regulatory variants affect expression only after stimulation activates the specific enhancer or transcription-factor program
Developmental state	Organogenesis and maturation impose time-limited regulatory programs through chromatin remodeling, DNA methylation, and isoform switching	Regulatory variants affect phenotype only during specific developmental windows
Sex and hormonal state	Hormone receptors and sex-biased regulatory programs redirect enhancer use, transcriptional co-regulation, and immune-metabolic signaling	The same variant can have different effects under different endocrine or sex-biased regulatory states
Metabolic and inflammatory state	Insulin resistance, nutrient stress, cytokine signaling, interferon activation, and mitochondrial stress reshape regulatory demand	Variants affecting these stress-response programs become more penetrant under metabolic or inflammatory stress
Disease-tissue state	Injury, treatment, fibrosis, cell-composition shift, and disease-interaction eQTLs alter the regulatory architecture of affected tissue	Variant effects measured in healthy tissue may disappear, reverse, or emerge in diseased tissue
Ageing and tissue-reserve state	Senescence, epigenetic drift, impaired proteostasis, mitochondrial dysfunction, and stem-cell exhaustion reduce buffering capacity	Inherited risk has greater clinical impact when repair capacity and physiological reserve decline

### Cellular activation state and gene regulation

Cellular state helps determine when genetic effects on gene regulation become manifested. Many variants identified by genome-wide association studies lie in noncoding regions rather than protein-coding sequence (Maurano et al., 2012). Fine-mapping studies have mapped candidate causal variants to enhancer activity, chromatin features, transcription factor binding, and gene expression (Farh et al., 2015). Because these regulatory mechanisms depend on tissue, cell type, and activation state, a variant may have little observable effect when the relevant regulatory elements are inactive, but a measurable effect when the corresponding pathway or stimulus-response program is active.

The same external exposure can correspond to different cellular states. In infection, immune cells may enter interferon-stimulated, inflammatory, exhausted, or resolving states. Genetic effects may differ across these states. In primary monocytes, interferon-γ or lipopolysaccharide stimulation can reveal expression quantitative trait loci (eQTLs) not detected at baseline (Fairfax et al., 2014), and enhancer priming can influence later immune responses (Alasoo et al., 2018).

Single-cell and disease-tissue studies support state-dependent genetic effects. In immune-mediated diseases, disease-associated variants often function in particular immune-cell populations and regulatory pathways rather than acting uniformly across all immune cells (Ota et al., 2021). Single-cell eQTL studies further show that genetic control of autoimmune disease-associated genes can differ by cell type (Yazar et al., 2022) and by T-cell state (Nathan et al., 2022). In human brain tissue, disease state can change allelic effects on gene expression, and merging diseased and non-diseased tissue can obscure genetic interpretation for central nervous system phenotypes (Haglund et al., 2025).

### Developmental state

Developmental state refers to stages of life in which tissues differ in gene-regulatory activity, endocrine milieu, immune maturity, and responsiveness to environmental exposure. A genotype-by-exposure effect may depend on the stage at which the exposure occurs. Early-life exposures can influence later disease risk by altering organ development, metabolism, immune function, and endocrine responsiveness (Gluckman et al., 2008; Hanson and Gluckman, 2014; Heindel et al., 2015). Genetic effects on gene expression and splicing have been mapped during mid-gestation in the developing human brain, and these fetal regulatory effects inform mechanisms of neuropsychiatric disease (Walker et al., 2019). For congenital structural defects involving G × E, genetic susceptibility and environmental perturbation must converge within specific windows of organogenesis, including neural tube closure, craniofacial patterning, and cardiac development (Krauss and Hong, 2016).

Puberty is a later developmental example. Activation of the hypothalamic-pituitary-gonadal axis changes sex steroid exposure, growth hormone and IGF-1 signaling, insulin sensitivity, and adipose distribution. Pubertal timing is highly polygenic and is linked to adiposity, hormone regulation, and growth (Day et al., 2017; Kentistou et al., 2024). Puberty is therefore not only a chronological boundary. Adiposity before puberty may reflect early growth and metabolic state, whereas adiposity during puberty also intersects with sex steroid production, insulin resistance, and reproductive-axis maturation. A genotype-by-adiposity or genotype-by-diet interaction may thus differ across pubertal stages because the exposure is embedded in different endocrine and metabolic pathways.

### Sex and hormonal state

Sex and hormonal state determine whether an exposure acts through sex-biased endocrine, metabolic, and immune pathways. Genetic effects may depend on sex-chromosome biology, gonadal hormone signaling, and sex-specific gene regulation (Khramtsova et al., 2019; Ober et al., 2008). Sex should therefore be modeled as a biological state when the exposure or phenotype is sex-dependent, rather than used only as an adjustment variable.

Sex shapes immune responses to self-antigens and foreign antigens, including innate immunity, adaptive immunity, infection response, and autoimmune risk (Klein and Flanagan, 2016). Female-biased autoimmunity is linked to stronger humoral and cellular immune responses, X-linked immune regulation, sex-hormone signaling, and sex differences in inflammatory pathways (Klein and Flanagan, 2016; Voskuhl, 2011). In type 1 diabetes, sex differences have been reported in incidence, age at onset, and clinical course across populations (Gale and Gillespie, 2001). Sex conditions genetic risk for type 1 diabetes through immune-cell behavior, hormone-responsive enhancers, X-escape gene dosage, sex-biased chromatin states, and polygenic load; sex may also alter how environmental factors such as vitamin D deficiency and enteroviral infection relate to genetic risk (Qu and Hakonarson, 2025).

### Metabolic and inflammatory state

Metabolic and inflammatory states condition genetic susceptibility through energy balance, insulin resistance, lipid handling, and immune activation. In type 2 diabetes, obesity and physical inactivity increase insulin resistance, but diabetes develops only when beta cells fail to meet the increased insulin demand. Variants affecting insulin secretion, such as *TCF7L2* rs7903146, may have limited clinical effect in insulin-sensitive adolescents but increase diabetes risk under insulin resistance, when higher metabolic demand exposes insufficient beta-cell compensation (Cropano et al., 2017). A genotype-by-activity association may therefore be conditioned by an insulin-resistant state.

In familial hemophagocytic lymphohistiocytosis, genetic defects in cytotoxic lymphocyte function can become clinically expressed as a cytokine storm syndrome when infection triggers uncontrolled immune activation, hypercytokinemia, and tissue injury (Canna and Behrens, 2012). Genetic susceptibility is thus conditioned by the infection-induced inflammatory state.

### Ageing and tissue-reserve state

Ageing and tissue reserve determine whether tissues can buffer genetic liability and environmental stress. Ageing is marked by progressive loss of physiological integrity, driven by interconnected processes such as genomic instability, telomere attrition, epigenetic alterations, loss of proteostasis, deregulated nutrient sensing, mitochondrial dysfunction, cellular senescence, stem-cell exhaustion, and altered intercellular communication (López-Otín et al., 2013). Geroscience frames these processes as shared drivers of chronic disease (Kennedy et al., 2014).

As these processes accumulate, tissue reserve declines and the regulatory state of the tissue changes. Ageing can change both baseline molecular programs and genotype-dependent regulatory effects. Transcriptomic studies show age-related shifts in biological programs associated with major diseases (Aramillo Irizar et al., 2018), while eQTL analyses show that genetic effects on gene expression can also vary with age (Bryois et al., 2017). Genetic variants may thus influence gene expression differently across ageing-related tissue states. The same genotype-by-exposure effect may have different consequences in a tissue with preserved repair capacity than in one shaped by senescence, inflammation, vascular injury, or reduced physiological reserve.

In rheumatoid arthritis, accelerated biological ageing and genetic predisposition interact, rather than acting independently, leading to higher disease risk (Chen et al., 2024). Across common age-related diseases, the relative contributions of genetic susceptibility and environmental exposure differ. In UK Biobank, genetic susceptibility contributed more to dementia and breast, prostate, and colorectal cancers, whereas environmental exposure contributed more to lung, heart, and liver diseases (Argentieri et al., 2025). A polygenic risk score (PRS) is stable across the life course, but its clinical impact changes as tissue reserve, metabolic state, and disease incidence change with age. A high type 2 diabetes PRS may have less near-term clinical relevance in childhood than in adulthood, although early metabolic effects may still be detectable.

## Methodological implications for state-aware genetic epidemiology

State-aware analysis defines what the exposure represents, identifies the biological state most likely to condition susceptibility, and tests whether that state accounts for the observed interaction. A preliminary algorithm is described in Supplementary Table S1 as a starting framework for future refinement.

### Classifying the exposure-state relation before testing interaction

Before fitting a genotype-by-exposure model, investigators should classify the exposure-state relation: upstream behavior, correlate or consequence of state, imperfect marker of internal dose or response, or trigger of state transition. Causal diagrams can specify whether the exposure is modeled as a cause of state, correlate of state, mediator between state and disease, or consequence of disease-related state (Moreno-Betancur et al., 2018).

When an exposure may reflect altered biological state, causal interpretation requires temporal protection. In G × E analysis, an exposure can appear to modify genetic risk when it is actually indexing early biological change in genetically susceptible individuals. Dietary restriction, reduced activity, medication use, or sleep change may follow preclinical disease, inflammation, pain, organ dysfunction, or fatigue. Rezende et al. show how reverse causation can bias lifestyle-mortality associations and why excluding early events or using lagged exposure definitions can test whether the exposure preceded disease rather than reflected early pathology (Rezende et al., 2022).

When an exposure incompletely measures internal dose or response, the interaction can be assigned to the wrong modifier. Reported diet may miss absorption, metabolism, nutritional status, or inflammatory response. Infection status may miss pathogen burden, interferon activity, immune-cell composition, or inflammatory resolution. Imprecise exposure measurement can distort interaction estimates and obscure the biological state that conditions susceptibility. The exposure should be calibrated against internal dose or response markers, and the interaction should be retested using those markers when available.

For exposures that trigger state transitions, a single baseline interaction term is usually insufficient. Infection, puberty, pregnancy, menopause, adipose expansion, tissue injury, and ageing-related stress can move individuals into states in which genetic susceptibility becomes expressed. Repeated measures, landmark designs, time-varying models, and before-after assessments test whether the genetic effect emerges after the state transition rather than being constant across exposure categories.

### Measuring biological state in G × E analysis

A biological-state variable should be valid for the mechanism being tested. It should represent the condition proposed to shape genetic susceptibility, with attention to spatial and temporal specificity. For gene-regulatory mechanisms, spatial specificity may require measurement in the relevant tissue or cell type, because many complex-disease variants act through regulatory programs that differ across tissues and cell populations (GTEx Consortium, 2020). Regulatory effects may also differ between resting and activated cells, or between healthy and diseased tissue. Measurement should therefore match the condition in which the variant is expected to act.

Temporal specificity matters because the same biological state can have different meanings depending on when it is measured. A biological state measured before exposure, after exposure, or after disease onset, treatment initiation, or behavioral change may represent different biological relations. Congenital structural defects require convergence between genetic susceptibility and environmental perturbation during restricted windows of organogenesis (Krauss and Hong, 2016). Genetic effects on gene expression can vary with age, as shown by age-dependent eQTLs (Bryois et al., 2017). Mediation-interaction methods can help separate temporal roles of biological state, including whether state functions as a mediator, modifier, or post-disease consequence, but require assumptions about temporal order and confounding (VanderWeele, 2016).

### Interpreting genotype-by-exposure signals through biological state

A genotype-by-infection signal should not be interpreted biologically until immune activation has been considered. If the genotype-by-infection signal weakens after interferon activity or inflammatory state is included, infection status may have indexed the immune condition that shaped genetic susceptibility. If the genotype-by-immune-state association is stronger or more coherent than the genotype-by-infection association, immune state is the more direct condition. Persistence of both signals may indicate that infection acts partly through immune function and partly through another pathway, such as receptor-mediated viral entry in COVID-19 (Hoffmann et al., 2020).

### Replication and prediction by biological-state comparability

Replication of a G × E signal should not be defined only by matching genotype, exposure, and phenotype categories. Cohorts with the same exposure category may differ in biological state. Such mismatch may contribute to poor replication of reported G × E signals. State-aware replication thus requires comparability of the conditions used to explain the interaction: timing, disease or treatment context, and relevant state measures. Many monocyte cis-eQTLs were detected only after interferon-γ (IFN-γ) or lipopolysaccharide stimulation, while a significant proportion of naïve-state cis-eQTLs were no longer detected after treatment (Fairfax et al., 2014). Replication of state-dependent genetic effects requires matching cellular state and timing, not only the variant and trait.

State-aware prediction of genetic risk should be interpreted within the biological states in which genetic risk is likely to become clinically relevant. A PRS estimates inherited liability, but its clinical meaning depends on the biological and population context in which risk is interpreted (Torkamani et al., 2018; Lewis and Vassos, 2020). Before the COVID-19 pandemic, genetic susceptibility to severe COVID-19 had no clinical meaning because the relevant viral exposure and host-response state were absent.

## Discussion

Genetic susceptibility is conditional on biological state. A genetic variant may remain latent, become functional, or lose clinical relevance depending on whether the relevant biological state is active. Disease arises when genetic risk operates within a permissive state. This framing accounts for state-dependent allelic effects on gene expression, poor replication of exposure-defined G × E signals across cohorts with different biological contexts, and the changing clinical meaning of polygenic risk across the life course.

Biological states shape disease biology across major medical fields. In oncology, adiposity-related metabolic and hormonal states may shape inherited breast cancer susceptibility: in *BRCA1/2* mutation carriers, adiposity was associated with DNA damage in normal breast epithelium and increased mammary tumor penetrance in *Brca1* mouse models (Bhardwaj et al., 2023). In infectious disease, infection-triggered immune activation can expose inherited defects in cytotoxic lymphocyte function, producing hemophagocytic lymphohistiocytosis (Canna and Behrens, 2012). In chronic metabolic disease, insulin-resistant state conditions the effect of TCF7L2 variation on type 2 diabetes risk by increasing demand for beta-cell compensation under metabolic stress (Cropano et al., 2017). In pediatric disease, organogenesis conditions folate-pathway genetic susceptibility to neural tube defects because genetic liability and folate-related perturbation must converge during neural tube closure (Krauss and Hong, 2016).
