# Supplementary material for: Genetic susceptibility shaped by biological state: beyond gene-environment interaction
Source: Front Genet. 2026 Jun 25;17:1877629. doi: 10.3389/fgene.2026.1877629 (PMC13345597; doi:10.3389/fgene.2026.1877629)
Supplement: Supplementary file 1 [file Table1.docx]

**Supplementary Table S1. State-aware approach for G×E interpretation**

| Step* | Self-question | Action | Example |
| --- | --- | --- | --- |
| 1 | Does the measured exposure directly represent the biological state expected to condition susceptibility? | Yes: model genotype × measured state, then proceed to Step 6.  No: proceed to Step 2. | Yes: insulin resistance measured by HOMA-IR.  No: physical activity used as a proxy for metabolic state. |
| 2 | Is the exposure likely to precede the biological state? | Yes: test whether exposure predicts later state and whether state modifies genetic effect; then proceed to Step 6.  No: proceed to Step 3. | Yes: inactivity preceding insulin resistance.  No: reduced activity after inflammatory symptoms begin. |
| 3 | Could the exposure reflect preclinical disease? | Yes: use lagged exposure definitions, early-event exclusion, repeated measures, or landmark analysis; then proceed to Step 6.  No: proceed to Step 4. | Yes: low meat intake from early kidney dysfunction.  No: long-term dietary pattern measured before disease onset. |
| 4 | Does the exposure measure internal dose or response? | Yes: prioritize internal dose or response markers in G×state models; then proceed to Step 6.  No: proceed to Step 5. | Yes: pathogen load or interferon activity.  No: infection status alone. |
| 5 | Does the exposure trigger transition into a new biological state? | Yes: use before-after, landmark, or time-varying models to test whether genetic effects emerge after transition; then proceed to Step 6.  No: retain exposure-defined interpretation with mechanistic uncertainty; if a candidate state can still be measured, proceed to Step 6. | Yes: infection triggering immune activation.  No: rural or urban residence without evidence of a biological-state transition. |
| 6 | Is the relevant state measured in the correct tissue, cell type, and time window? | Yes: test genotype × state using the measured state; then proceed to Step 7.  No: avoid mechanistic overinterpretation and improve state measurement before replication or translation. | Yes: stimulated immune-cell eQTLs after activation.  No: blood marker used for a disease-tissue regulatory state. |
| 7 | Is the exposure-state relation biologically coherent? | Yes: proceed to Step 8 to test whether biological state conditions the observed G×E association.  No: report that the biological state underlying the exposure-defined interaction remains mechanistically unresolved. | Yes: poverty-related T2D risk may operate through insulin resistance shaped by food insecurity and chronic stress.  No: a smoking-immune activation relation in obesity without inflammatory evidence or metabolic dysfunction. |
| 8 | Does biological state condition the observed G×E association? | Yes: estimate and interpret the G×E association within strata of biological state.  No: retain the G×E model and report that state-conditioning was not demonstrated. | Yes: high T2D PRS and poverty increase diabetes risk mainly under insulin resistance, whereas the same PRS and poverty may not translate into diabetes under heavy physical labor and calorie insufficiency.  No: G×E is observed, but measured metabolic-state markers do not modify the genetic effect. |
| 9 | Do replication cohorts share the same biological state? | Yes: compare effect direction and magnitude.  No: treat non-replication as potentially due to state mismatch. | Yes: same stimulation protocol and timing.  No: baseline cells compared with stimulated cells. |
| 10 | Is the genetic effect clinically relevant in the observed state? | Yes: interpret variant effects or PRS within that state.  No: report that clinical relevance in the observed state is not established; evaluate other states, longer follow-up, or standard risk models. | Yes: type 2 diabetes PRS under insulin resistance.  No: type 2 diabetes PRS in an insulin-sensitive child. |

* The steps are intended as a sequential decision tree to be followed in order. Step 1 is a branch point that determines whether the exposure already measures the relevant biological state; if not, Steps 2-5 classify how the exposure relates to state. Step 6 evaluates whether the state is measured at the correct biological scale. Steps 7–8 evaluate whether the exposure-state relation is biologically coherent and whether biological state conditions the observed G×E association. If the answer is “No” at Step 6 or Step 7, mechanistic interpretation should stop until state measurement or the exposure-state hypothesis is improved. Steps 9-10 are common concluding steps for replication across comparable biological states and clinical interpretation.
